# Oral oxycodone versus sublingual buprenorphine for postoperative pain control after pelvic exenteration (PROSPER): a pilot, registry-embedded, multi-centre, double-blind, placebo-controlled, randomised controlled trial

**DOI:** 10.1136/bmjopen-2026-117594

**Published:** 2026-06-22

**Authors:** Charlotte Johnstone, Cherry Koh, Stephanie Mathieson, Daniel Steffens, Kate White, Paul Gray, Craig Harris, Alexander Heriot, Kaitlin Kramer, Chi Kin Law, Chung-Wei Christine Lin, Xiaoqiu Liu, Gustavo Machado, Andrew McLachlan, Jonathan Penm, Bernhard Riedel, Tarik Sammour, Robert Sanders, Michael Solomon, Lilian Whitehead, Des Winter, Jamie Young, Asad Patanwala

**Affiliations:** 1 Department of Anaesthesia, Royal Prince Alfred Hospital, Sydney, New South Wales, Australia; 2 Sydney Pharmacy School, Faculty of Medicine and Health, University of Sydney, Sydney, New South Wales, Australia; 3 Faculty of Medicine and Health, University of Sydney, Sydney, New South Wales, Australia; 4 Surgical Outcomes Research Centre, Royal Prince Alfred Hospital, Sydney, New South Wales, Australia; 5 Sydney School of Public Health, Faculty of Medicine and Health, University of Sydney, Sydney, New South Wales, Australia; 6 Sydney Nursing School, Faculty of Medicine and Health, University of Sydney, Sydney, New South Wales, Australia; 7 The University of Queensland, Brisbane, Queensland, Australia; 8 The Royal Brisbane & Women's Hospital, Brisbane, Queensland, Australia; 9 Division of Cancer Surgery, Peter MacCallum Cancer Centre, Melbourne, Victoria, Australia; 10 The Sir Peter MacCallum Department of Oncology, The University of Melbourne, Melbourne, Victoria, Australia; 11 NHMRC Clinical Trials Centre, The University of Sydney, Sydney, New South Wales, Australia; 12 Institute for Musculoskeletal Health, Sydney Local Health District, Sydney, New South Wales, Australia; 13 The George Institute for Global Health, University of New South Wales, Sydney, New South Wales, Australia; 14 Department of Pharmacy, Prince of Wales Hospital, Sydney, New South Wales, Australia; 15 Department of Critical Care, The University of Melbourne, Melbourne, Victoria, Australia; 16 Department of Surgery, Royal Adelaide Hospital, Adelaide, South Australia, Australia; 17 Faculty of Health and Medical Sciences, University of Adelaide, Adelaide, South Australia, Australia; 18 Department of Colorectal Surgery, St Vincent’s University Hospital, Dublin, Leinster, Ireland; 19 Department of Anaesthesia, Perioperative Medicine, and Pain Medicine, Peter MacCallum Cancer Centre, Melbourne, Victoria, Australia; 20 Department of Pharmacy, Royal Prince Alfred Hospital, Camperdown, New South Wales, Australia

**Keywords:** Cancer Pain, Drug Therapy, PAIN MANAGEMENT, Pelvic Pain, Colorectal surgery, Prospective studies

## Abstract

**Objectives:**

This study aims to estimate the rate of recruitment of participants.

**Design:**

This is a pilot, multicentre, double-blind, placebo-controlled, randomised controlled trial of oral oxycodone and sublingual placebo vs sublingual buprenorphine and oral placebo for postoperative pain management for 7 days after pelvic exenteration.

**Setting:**

Patients will be recruited from three metropolitan quaternary referral centres that offer advanced gastrointestinal surgery in Australia.

**Participants:**

The inclusion criteria will be patients over the age of 18 years undergoing pelvic exenteration surgery and exclusion criteria are previous adverse events related to the study drugs, currently requiring monoamine oxidase inhibitor medications and if epidural analgesia is planned in the perioperative period.

**Interventions:**

Enrolled patients will undergo pelvic exenteration surgery and be initiated postoperatively on patient-controlled analgesia. In the postoperative period, when clinically appropriate to take oral medications, patients will be commenced on trial analgesia for 7 days. Participants will be randomised to receive either oral active oxycodone 5–10 mg up to 3 hourly as required (with sublingual placebo) or sublingual active buprenorphine 200–400 mcg 3 hourly as required (with oral placebo).

**Main outcome measures:**

The primary outcome measure is the rate of recruitment over a 6-month period. Secondary outcomes include an assessment of missing data, protocol adherence and acceptability of the trial to participants.

**Ethics and dissemination:**

The trial received ethics approval from Sydney Local Health District, Royal Prince Alfred Hospital Human Research Ethics Committee (No: X25-0128 & 2025/ETH01058). The results of the study will be disseminated by publication and presentation at local annual scientific meetings in Australia.

**Trial registration number:**

The study protocol is prospectively registered at the Australian New Zealand Clinical Trials Registry (ANZCTR) (www.anzctr.org.au; ACTRN12625000901404).

**STRENGTHS AND LIMITATIONS OF THIS STUDY**

- The strengths of this study are that it is a placebo-controlled design where the participants, researchers and treating specialists do not know which intervention has been allocated.
- The outcomes include patient-reported outcome measures and clinically meaningful analgesic measures.
- Consumers have been involved in defining the outcomes that they considered to be important, and this study may apply to all patients undergoing major abdominal surgery.
- A potential limitation is variability in clinicians’ decisions to transition patients from patient-controlled analgesia to oral or sublingual analgesia.

## Introduction

 Pain and its management following major abdominal surgery are burdensome for patients. The adverse outcomes from opioid analgesia include delayed nutrition,^1 2^ sleep disturbance,^3^ cognitive dysfunction,^4^ respiratory depression, poor cancer outcomes and persistent opioid use and dependence.^5^ For example, in Australia, of those patients who had abdominal surgery and were discharged on opioid analgesia, 5.9% still required ongoing opioid analgesia 3 months following surgery.^6 7^ The adverse effects of opioid analgesia are especially problematic for patients undergoing pelvic exenteration surgery. Patients having pelvic exenteration require opioid analgesia for several weeks as inpatients and frequently for prolonged periods as outpatients.

Pelvic exenteration is a major abdominal surgery primarily performed to treat pelvic cancer.^8^ To obtain clear resection margins (all tumour removed) from advanced or recurrent pelvic malignancies, surgical removal of bowel and other organs such as the bladder and sex organs is required. Other structures such as sacrum, nerves and the pelvic floor may also need to be removed. It is a complex, life-changing surgery that can take 8–12 hours. Following pelvic exenteration, major complications such as severe pain,^9^ delayed gastrointestinal activity,^10^ infection^11^ and delayed wound healing^10^ are present in 37.8% of patients.^12^ Currently, pain following pelvic exenteration surgery is managed with a variety of techniques, including spinal analgesia with intrathecal morphine, epidural analgesia,^13^ wound catheters,^14^ patient-controlled analgesia^15^ and oral analgesic agents, including opioids^14^ and other agents such as paracetamol, non-steroidal anti-inflammatory agents and anti-neuropathic agents. Despite these techniques, the extent of surgery and ongoing surgical complications can make pain management in the perioperative period challenging.^14^

Clinical guidelines, such as those from the Australian and New Zealand College of Anaesthetists, recommend that analgesic stewardship is practised before, during and after surgery in support of facilitating improved patient outcomes.^16^ Analgesic stewardship considers analgesic drugs and adjuvant medications to strive for a balance between pain relief that is sufficient to facilitate patient recovery and restoration of function and minimisation of analgesia-related harms to the patient and others.^16^ This principle is relevant for pelvic exenteration surgery. Our previous research in patients undergoing pelvic exenteration surgery demonstrated that compliance with a pain management algorithm, including regional analgesia and preferentially prescribing tapentadol and buprenorphine, reduced the oral morphine equivalent daily dose on discharge.^14^ However, studies comparing different opioid analgesic agents or regional analgesia techniques and their analgesic and adverse outcomes in this population are limited.

The use of ‘multimodal analgesia’ is needed for treating postoperative pain effectively.^17^ This has been described as achievement of sufficient analgesia due to additive or synergistic effects between different analgesics, with concomitant reduction of side effects, due to resulting lower doses of analgesics and differences in side effect profiles.^17^ Internationally, surgical societies have incorporated the concept of multimodal analgesia, including local anaesthetic agents, non-opioid analgesics and opioid analgesic agents, into Enhanced Recovery After Surgery (ERAS) guidelines, a multimodal care pathway designed to achieve early recovery for patients following major surgery.^18^ The PelvEx Collaborative is an international group that aims to improve pelvic exenteration care by collecting data and creating guidelines. PelvEx has aligned with ERAS and created guidelines regarding perioperative care for patients undergoing pelvic exenteration.^19^

This study will use a multimodal analgesia approach embedded in the perioperative pathways, including combinations of intraoperative spinal analgesia with intrathecal morphine, regional blocks using postoperative abdominal wall local anaesthesia infusions, patient-controlled analgesia of an opioid, oral or sublingual opioid analgesia and other oral analgesic adjuncts, for example, paracetamol, non-steroidal anti-inflammatory drugs (NSAIDs) and antineuropathic agents.

It is not clear which oral or sublingual opioid analgesic agent provides the best analgesia to adverse effect profile in the postoperative period. Oxycodone is a commonly prescribed opioid analgesic agent in the postoperative period in Australia, accounting for more than 80% of opioids prescribed for some procedures.^20^ One potential alternative strategy is to consider low-dose sublingual buprenorphine. In addition to being an opioid receptor full agonist, buprenorphine is a kappa receptor partial agonist.^21^ In terms of analgesia, buprenorphine behaves as a full agonist and provides analgesia equivalent to other opioids such as morphine and fentanyl.^22^ Buprenorphine may help reduce hyperalgesia because opioid-induced hyperalgesia is partly driven by dynorphins activating kappa opioid receptors, and buprenorphine’s antagonism at these receptors can counteract this effect. This mechanism may also help reduce opioid tolerance, as shown in a pre-clinical model.^23^ The partial agonism seen in vitro with buprenorphine may be beneficial for gastrointestinal activity. The sublingual presentation of buprenorphine has further benefits for patients who are nil-by-mouth.

A comparison of the analgesic and adverse effect outcomes of oxycodone versus buprenorphine has been performed by Heldreich *et al*.^24^ They completed a retrospective cohort study of patients following elective and emergency abdominal surgery (not including pelvic exenteration surgery) who were prescribed oral oxycodone analgesia compared with another cohort who were prescribed sublingual buprenorphine analgesia.^24^ Those patients who transitioned to sublingual buprenorphine had a clinically significant reduction in oral morphine equivalent daily dose of 26 mg on day two following surgery.^24^ However, sublingual buprenorphine has not been compared with oral oxycodone in a randomised controlled trial of patients undergoing major abdominal surgery.

The primary objective of this pilot study is to determine the feasibility of conducting an adequately powered definitive clinical trial. Secondary objectives include identifying the extent of missing data used for primary and secondary outcome measures that will be used for a full-scale trial, protocol adherence and acceptability of the study by participants.

## Methods and analysis

### Study design

The study will be conducted in three quaternary referral public hospitals in Australia, which have well-established advanced gastrointestinal surgical units. These include the Royal Prince Alfred Hospital in Sydney, the Peter McCallum Cancer Centre in Melbourne and the Royal Adelaide Hospital in Adelaide. Participant recruitment for the study commenced on 7 April 2026, and data collection is anticipated to continue until November 2026.

The trial protocol was approved by the Sydney Local Health District (Approval number: X25-0128 & 2025/ETH01058).

### Participants and recruitment

Participants will be recruited from patients who are referred to surgical clinics of each hospital for consideration of pelvic exenteration surgery. Participants will be given a participant information and consent form and a one-page information brochure (online supplemental consent form S1, online supplemental brochure S2). Study surgeons will obtain a medical history, including current treatments and medications, and screen the patient for eligibility. Eligible participants will meet all the following criteria:

- Undergoing pelvic exenteration surgery for advanced primary or recurrent pelvic (eg, rectal, bladder, prostate, uterine and other) malignancies.
- Adults≥18 years.

Patients will be excluded if they meet any of the following criteria:

- If the treating clinician does not consider both study drugs (oral oxycodone or sublingual buprenorphine) to be a suitable option for the patient or does not consider the trial to be in the best interests of the patient based on precautions with study drugs or previous adverse events attributed to study drugs.
- If they require monoamine oxidase inhibitors within 14 days of planned surgery. These include phenelzine, tranylcypromine, moclobemide, selegiline, rasagiline and linezolid. This is because of potential drug interactions with opioids resulting in serotonin syndrome.
- The treating team intends to use epidural analgesia perioperatively or if epidural analgesia is used before randomisation. This is because the postoperative management and opioid consumption is different in the few patients who receive epidurals.
- If a person is unable to provide informed consent.

If a patient is eligible, they will be referred by the clinic to a researcher to obtain consent. The research team will collect baseline data by phone or face-to-face. Then the participant will proceed to the planned surgery.

### Randomisation and blinding

A blinded, independent trial statistician will create a permuted block randomisation schedule using R (V.4.3.1 or above), which will then be supplied to the drug manufacturer (Syntro Health, Richmond, Australia), for preparation of the blinded medication packs. Randomisation will occur on postoperative day 1. Participants will be randomly assigned 1:1 to the buprenorphine group or the oxycodone group. Placebos will be identical looking medication compared with their oral oxycodone and sublingual buprenorphine counterparts and will also be supplied by Syntro Health. Each blinded medication pack will include a randomisation number, corresponding to the randomisation schedule. On receipt of the prescription postoperatively for study medications, the investigational drug service at the hospital pharmacy will dispense study medications based on the sequentially numbered kit. Participants, treating clinicians and researchers will be blinded to group allocation.

### Study intervention

When the treating clinician considers that the participant is ready to transition from intravenous patient-controlled analgesia to oral analgesia, the investigational analgesic agents are prescribed by the treating physician and dispensed. Participants will receive either oral active oxycodone 5–10 mg up to 3 hourly as required (with sublingual placebo) or sublingual active buprenorphine 200–400 mcg 3 hourly as required (with oral placebo) when they request analgesia. The term ‘as required’ means that doses will be administered only when patients request it for pain control. The amount of intervention medication that may be administered is 16 tablets per 24 hours. These doses and time intervals are based on the investigators’ clinical experience with this cohort of patients and what was being used across the hospitals in routine practice. The doses selected are also considered to be equianalgesic as defined by the Faculty of Pain Medicine, Australian and New Zealand College of Anaesthetists opioid conversion ratios.^25^

**Figure 1:**
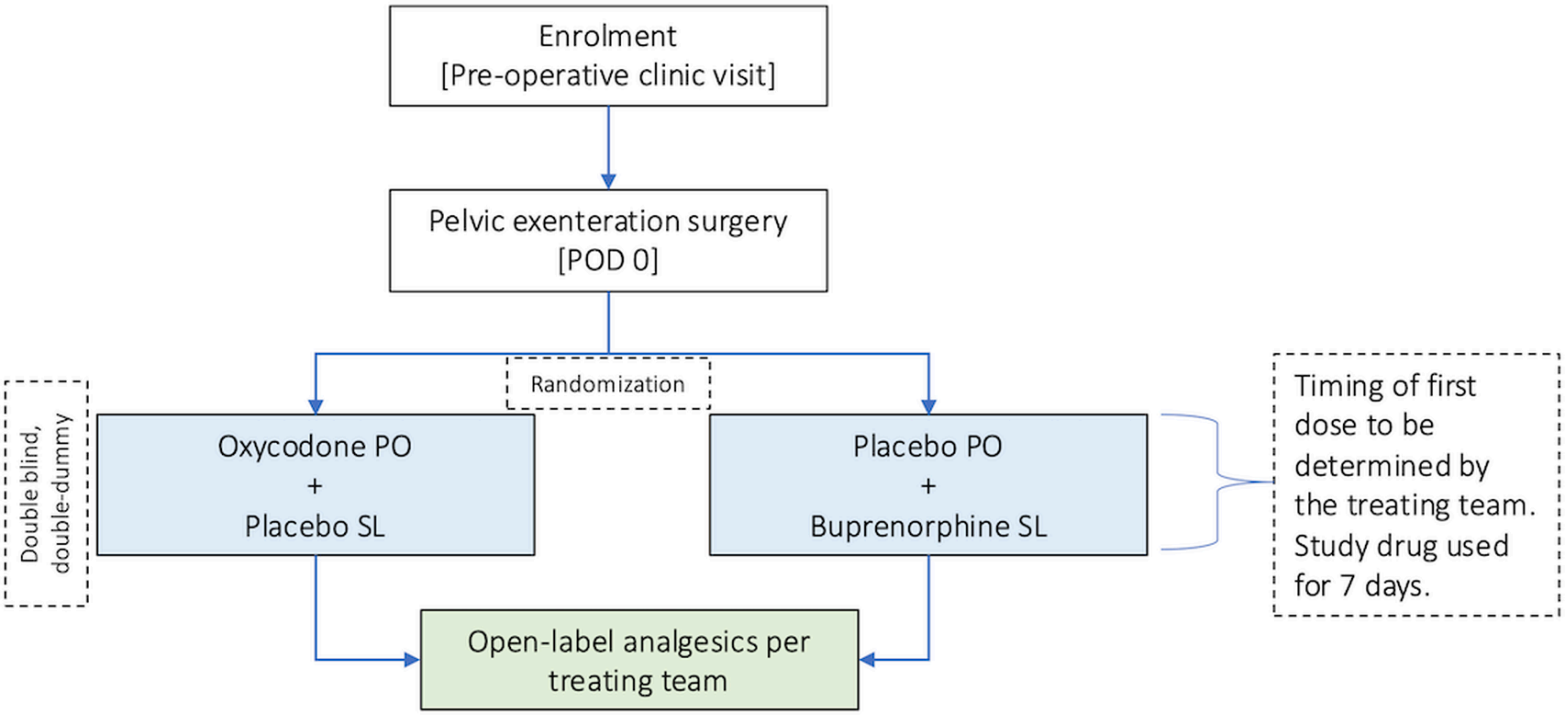
Study flow chart of interventions for participants. POD, postoperative day. PO, oral. SL, sublingual.

A study flow chart of the intervention is in figure 1. The specifications of the interventional medical products oxycodone, buprenorphine and their placebos are described in table 1.

**Table 1:** The specifications of the interventional medical products

	Oxycodone	Oxycodone placebo	Buprenorphine	Buprenorphine placebo
Colour	White	White	White	White
Shape	Round	Round	Round	Round
Mark	0.5	0.5	L	L
Score	05	05	Nil	Nil
Dimensions	10×3.7 mm	10×3.7 mm	5.56×5.56 mm	5.56×5.56 mm
Opioid	Oxycodone	Nil	Buprenorphine	Nil
Excipients	Stearic acid, microcrystalline cellulose, lactose	Stearic acid, microcrystalline cellulose, lactose	Lactose monohydrate, mannitol, maize starch, povidone, magnesium stearate, citric acid, sodium citrate dihydrate	Lactose monohydrate, mannitol, maize starch, povidone, magnesium stearate, citric acid, sodium citrate dihydrate

Analgesia management may be escalated as per clinical need and usual practice. Pain is measured by the nursing staff through pain scores or functional activity. For example, as required parenteral analgesia such as subcutaneous morphine may be prescribed for severe pain that is not managed with the trial medication.

The suggested approach for all patients is to receive non-opioid analgesics, such as paracetamol, to minimise the use of opioids. Additional analgesics for refractory pain, for example, morphine, tapentadol, methadone, tricyclic antidepressants, gabapentinoids and ketamine, are permitted to manage pain as required in addition to the trial medications as part of a pragmatic approach to mimic usual care. The use of additional analgesics will be collected as study data and considered part of usual care and not a protocol deviation. After 7 days of investigational analgesic use, patients will receive open-label usual analgesia care as prescribed by their treating clinician. The intervention is outlined in figure 1.

### Data collection and management

Data collection will be performed by trained study researchers preoperatively and on postoperative days 1–14. A standardised case record form will be used across all hospitals. Data will be obtained from medical records and patient questionnaires. In addition, a study doctor (CJ) will conduct qualitative interviews (online supplemental interview guide S3), pain questionnaires and quality of life surveys on postoperative day 90. Questionnaires during hospitalisation will be conducted face-to-face, whereas postoperative day 90 data will be collected either face-to-face, electronically or by phone by trained researchers. All other data will be obtained from the electronic medical records. PelvEx registry data will be supplied by the respective hospitals.

### Outcomes

**Figure 2:**
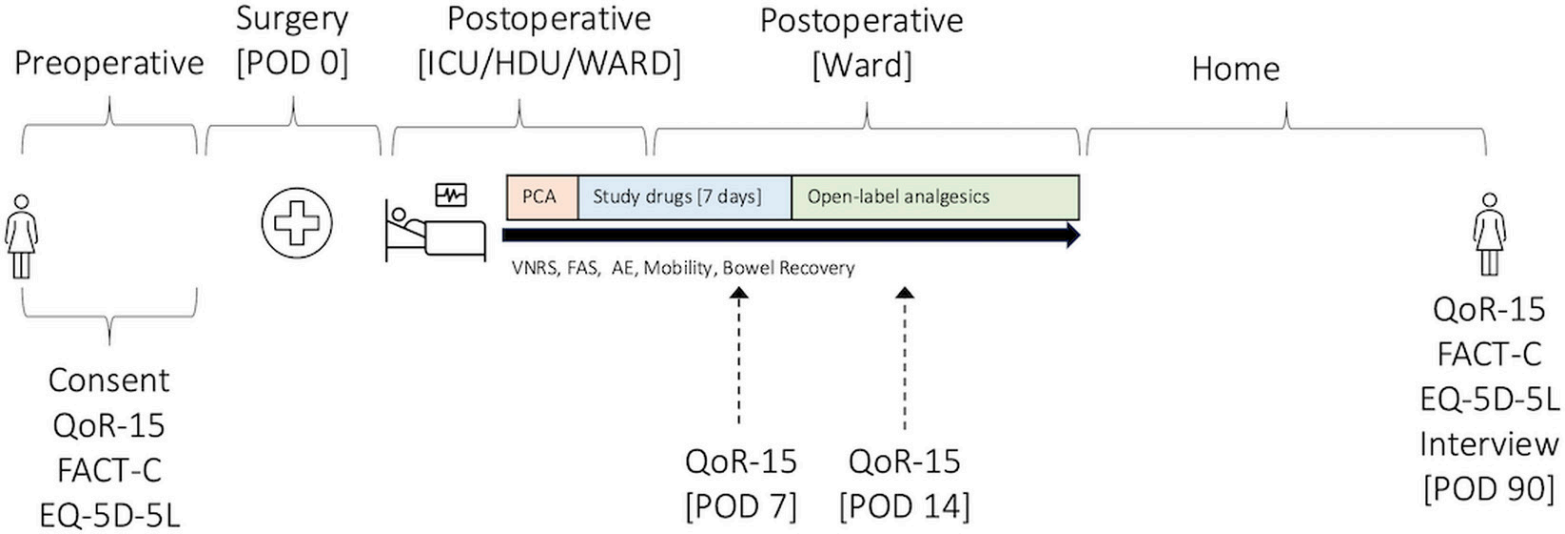
Figure outlining participant consent, surveys and follow-up. AE, adverse events; EQ-5D-5L, European Quality of Life Five-Dimensional Five-Level Questionnaire; FACT-C, functional assessment of cancer therapy-colorectal; FAS, Functional activity scale; HDU, high dependency unit; ICU, intensive care unit; POD, post-operative day; QoR-15, Quality of Recovery-15; VNRS, Verbal Numeric Rating Scale; Ward, inpatient postoperative care

The schedule of enrolment and assessments is outlined in table 2. Figure 2 outlines participant consent, surveys and an interview schedule.

**Table 2:** Schedule of enrolment and assessments for participants in PROSPER

Assessments Day	Enrolment	POD 1–6	POD 7	POD 8–13	POD 14	POD 90
Participant consent	**✓**					
Inclusion/exclusion criteria	**✓**					
Demography and medical history	**✓**					
Surgery and hospitalisation information		**✓**	**✓**	**✓**	**✓**	
Postoperative analgesics		**✓**	**✓**	**✓**	**✓**	
QoR-15	**✓**		**✓**		**✓**	**✓**
VNRS	**✓**	**✓**	**✓**	**✓**	**✓**	
FAS	**✓**	**✓**	**✓**	**✓**	**✓**	
Mobility assessment	**✓**	**✓**	**✓**	**✓**	**✓**	
Bowel recovery		**✓**	**✓**	**✓**	**✓**	
Adverse event assessment		**✓**	**✓**	**✓**	**✓**	
Blinding assessment					**✓**	
Patient interviews						**✓**
Quality of life (EQ-5D-5L^*^, FACT-C^*^)	**✓**					**✓**

* Obtained from PelvEx registry.

EQ-5D-5L, European Quality of Life Five-Dimensional Five Level Questionnaire; FACT-C, Functional Assessment of Cancer Therapy-Colorectal; FAS, Functional Activity Scale; POD, postoperative day; QoR-15, Quality of Recovery-15; VNRS, Verbal Numeric Rating Scale.

### Primary feasibility outcome

The proportion (or number) of total eligible patients undergoing pelvic exenteration who are randomised over a 6 month recruiting time frame. The threshold for this outcome is≥30% of all patients receiving pelvic exenteration surgery≥30 randomised participants at 6 months, whichever is higher. The rate of recruitment is important for this trial as it would ensure that an adequate sample size can be achieved for a definitive trial based on the number of pelvic exenterations conducted across the included hospitals in Australia. This rationale is further detailed in the sample size section.

### Secondary feasibility outcomes

- Quality of Recovery-15 (QOR-15)^26^: The proportion of randomised participants with no missing values on QoR-15 measure on postoperative day seven and day 14. A target is set for ≥80% complete data for each time point.
- Bowel recovery: The proportion of randomised participants with no missing values for each of the following metrics: time to first bowel movement and time to first solid diet. A target is set for ≥80% complete data for each metric.
- Pain scores: The proportion of randomised participants with no missing values on the Verbal Numerical Rating Scale^27^ and Functional Activity Scale^28^ up to postoperative day seven. A target is set for ≥80% complete data for each.
- Protocol adherence: The proportion of randomised participants who receive cross-over open-label treatment or comparator drugs during the 7 days of study. A target is set for≤20% of participants with cross-over to other study drugs.

### Other data collected

- Demographic data and medical history will include participants’ age, sex (at birth), height, weight, reason for surgery and preoperative analgesic agents will be collected at baseline.
- Surgery and hospital data will include the surgical margin,^29^ surgical time hours, blood transfusion required (Y/N), perioperative analgesic agents, occasions of returns to the operating theatre, day of extubation, presence and location of abdominal catheters, intensive care unit (ICU) length of stay (hours), occasions of ICU admission, hospital length of stay (days), number of emergency department visits and re-hospitalisations after hospital discharge up to day 90.
- Postoperative analgesia (opioids and non-opioids) that are administered postoperatively by any route at baseline and daily on postoperative days 1–14.
- Mobility will be measured using the Johns Hopkins Highest Level Mobility Scale,^30^ and will be assessed at baseline and daily on days 1–14 following surgery.
- Quality of life using the EuroQol 5-Dimension 5-Level Health Survey (EQ-5D-5L),^31^ collected at baseline and on postoperative day 90.
- Functional quality of life using the Functional Assessment of Cancer Therapy-Colorectal (FACT-C).^32^ This instrument is specifically validated for colorectal cancer patients. This survey will be obtained from sites participating in the PelvEx registry at baseline and postoperative day 90.
- All adverse events will be collected daily on days 1–14 following surgery or until discharge from the hospital. Due to the high frequency of surgical complications following pelvic exenteration surgery, only serious adverse drug reactions (ADR) will be reported. ADRs will only be reported after the first dose of the study drug is administered and up to 24 hours after the last dose is administered. The most common ADR due to the trial medications is nausea and vomiting. More serious adverse events (SAE) related to ADR resulting in an event that is life-threatening or results in death will be reported immediately to the serious events committee (AEP, CJ, SM, DS, KW, CK). This committee will review the event and will consider unblinding the treatment allocation as required. The Sydney Local Health District ethics committee will be informed of the SAE. Serious surgical complications will also be collected.
- A blinding assessment will be conducted on post-operative day 14. Participants will be asked to guess if they were randomised to oxycodone or buprenorphine (‘What study group do you think you were assigned to?’)
- In addition to the quantitative data above, participant experience will be explored guided by hermeneutic inquiry. In-depth semi-structured interviews will be conducted with participants and audio-recorded 3 months after surgery. The interviews will explore participants’ experience of the trial process (eg, recruitment experience, consent, acceptability of the interventions and assessments) and perspectives of post-operative pain management.

### Data integrity and analysis

All data collected for this study will be treated as confidential and stored in the University of Sydney REDCap database built specifically for this project. Data will be collected and entered into REDCap by designated research staff. The database will be monitored on an ongoing basis throughout the trial by the trial coordinator or the Coordinating Principal Investigator. Designated investigators from each site will be given access to REDCap for the data collected at their site. Participant interview recordings from postoperative day 90 will be recorded via Zoom and stored on the University of Sydney Research Data Store. Auditing of the trial will be performed by the principal investigator and the trial coordinator every month. Due to the small sample size, a Data Monitoring Committee will not be used during this trial. Additionally, both arms use Therapeutic Goods Administration-approved medications that are already part of the usual care in this patient population. There is no plan to obtain consent for further ancillary studies.

Interview transcripts will be transcribed verbatim and imported into NVivo V.20 for qualitative analysis. Using an inductive approach, the transcripts will be systematically coded by two experienced qualitative researchers. Following review of individual codes and reaching initial agreement, the two researchers will group codes into potential themes. These themes will be reviewed and confirmed by a third member of the research team.

### Sample size

In a definitive trial, we plan to use the primary outcome of QOR, as assessed by the QoR-15 at postoperative day 7. This will be a superiority study. Based on the minimal clinically important difference (MCID) on this instrument (MCID=8),^33^ common standard deviation (SD=18), we estimated that the full-scale trial would need 180 participants, based on a two-sided alpha of 0.05 and a power of 80%, considering 10% dropouts. Currently, the total number of pelvic exenterations conducted annually across all the three sites is also 180. Recruitment may start at different times at each site; to incorporate this, we have estimated that 30% of total pelvic exenteration patients recruited or≥30 participants over 6 months, whichever is higher, would enable us to conduct a full trial in the future.

### Statistical analysis plan

All analyses for primary and secondary feasibility outcomes are descriptive and will be reported as proportions (n/N, %). Trends in recruitment rates will be depicted visually across sites. Recruitment may start at different times at each site. The rate calculation will incorporate this differential timing. This value will also be stratified by site. We will explore outcomes for the planning of the full-scale trial. This will include estimating mean differences with 95% CI on the QoR-15. We expect that the composite score of the QoR-15 will be normally distributed. The SD of this score will be evaluated in the context of previous investigations and for planning a full-scale trial. The following variables will be considered as potential stratification variables in a full-scale trial based on our experience: 1. Preoperative opioid use, 2. Resection of bone or nerves. If there are meaningful differences in QoR-15 based on these variables and per our interviews with participants, then a stratified randomisation will be considered if we progress to a full-scale future trial. No imputation will be conducted for missing data, as no inferential testing is being conducted. There is no plan for an interim analysis.

In addition to the statistical analysis of primary and secondary outcomes, a value of information (VOI) analysis will be conducted. This analysis will integrate pilot trial data into a decision-analytic model (such as a decision tree or Markov model) to quantify the value of a definitive trial in producing evidence for clinical practice. The VOI analysis provides an economic justification for further research. Rather than proceeding with a costly definitive trial based on intuition, this approach quantifies whether the potential improvements in future patient outcomes and healthcare efficiency, gained from better evidence, justify the trial’s expense. It ensures the efficient allocation of limited research funds by prioritising studies with the greatest potential to enhance clinical decision-making and patient care.^34^ Aligned with standard cost-effectiveness methodology, participants’ quality of life will be assessed during follow-up using the FACT-C instrument and converted to health utilities (SF-6D) via a validated algorithm.^35^ Due to uncertainty regarding potential missing FACT-C data in the PelvEx registry, the EQ-5D-5L will also be collected as an alternative measure. Data on resource use, including drug costs, postoperative complications, severe adverse effects, critical care utilisation and hospitalisations, will be extracted from study records. National unit costs for all relevant healthcare services and events will be obtained from the annual inpatient fractions costing review. These individual expenses will be categorised into ‘cost buckets’ (eg, staffing, operating room and intensive care) for detailed analysis.

The decision model will be populated with data on costs, utilities (derived from FACT-C or EQ-5D-5L) and clinical events from the pilot trial. Monte Carlo simulation will be performed to characterise uncertainty in the model’s cost-effectiveness results and to compute key VOI metrics, such as the expected value of perfect information (EVPI). The EVPI estimates the maximum value of eliminating all current decision uncertainty. If the population-level EVPI exceeds the estimated cost of the definitive trial, further research is likely to be a cost-effective investment. The findings of this analysis will be reported in accordance with the Consolidated Health Economic Evaluation Reporting Standards 2022 (CHEERS 2022).^36^

### Patient and public involvement

People with lived experience consumer group were invited and provided one-on-one guidance to introduce them to the project and provide information to maximise their contributions, while ensuring a safe, sensitive and respectful environment for their involvement. Age, gender and cultural background were considered to ensure that the consumer group is diverse and representative of patients having this type of surgery. Consumers have been meaningfully involved since the study idea was conceived, and their input has guided the study design. This includes the selection of potential outcomes that are important to patients, development of patient brochures and participant information sheets. For example, in addition to pain, the ability to have their first meal was identified as important to consumers.

## Discussion

This study is the description of the pathway to conduct a pilot randomised controlled trial of oxycodone vs buprenorphine following pelvic exenteration. It is a study that targets feasibility outcomes such as recruitment rate, completion of data collection and acceptability of the trial to participants. Our primary outcome of recruitment rate is important to ensure the feasibility of a future definitive trial. If the recruitment is slower than anticipated, we would gain insight about this for future investigations in this population. The inclusion of secondary outcomes such as QOR-15, bowel activity and participant functional activity are important measures for a definitive future trial. One of the advantages of this trial is that participants, treating clinicians, investigators and statisticians are blinded to the investigative analgesic agents. The other advantage is that trained study researchers will enhance data quality and consistency.

The study results will be submitted for publication in scientific journals and presented at scientific meetings. Ultimately, we expect that we will understand if sublingual buprenorphine confers improved perioperative patient outcomes, such as enhanced functional activity and limited adverse effects compared with oxycodone following pelvic exenteration surgery.

Long-term, we hope that the results of this study will guide the opioid analgesic choices of clinicians who are involved in the pain management care of patients following pelvic exenteration surgery.

## Ethics and dissemination

The main ethical and safety considerations relate to the risk of adverse effects of the trial medications. Potential benefits include a reduced risk of adverse events from buprenorphine compared with oxycodone. There is also the potential for coercion as the clinicians and researchers may have dual roles and patients may feel obligated to participate. The potential for coercion has been minimised by separation of these roles as much as possible.

The trial results will be communicated with participants via email or mail based on their preference. It will be presented at local and national scientific meetings as an abstract/poster. The final manuscript will be published in a peer-reviewed journal. A final report will also be submitted to The Medical Research Future Fund and the University of Sydney, as the trial sponsor.

## Supplementary material

10.1136/bmjopen-2026-117594 online supplemental file 1

10.1136/bmjopen-2026-117594 online supplemental file 2

10.1136/bmjopen-2026-117594 online supplemental file 3

10.1136/bmjopen-2026-117594 online supplemental file 4
