# Supplementary material for: Oral oxycodone versus sublingual buprenorphine for postoperative pain control after pelvic exenteration (PROSPER): a pilot, registry-embedded, multi-centre, double-blind, placebo-controlled, randomised controlled trial
Source: BMJ Open. 2026 Jun 22;16(6):e117594. doi: 10.1136/bmjopen-2026-117594 (PMC13289154; doi:10.1136/bmjopen-2026-117594)
Supplement: online supplemental file 1 [file bmjopen-16-6-s001.pdf]

About the PROSPER trial

The **PROSPER** trial is comparing two opioid medicines that are used after surgery for pain control.

Both medicines are approved for use in Australia.

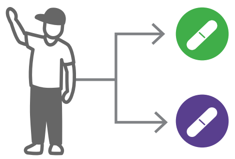

Oxycodone

Buprenorphine

> If you decide not to participate you may still get these medications as part of usual care.

PROSPER is a feasibility study, which means the aim is to determine if a future large trial is possible.

> **PROSPER** is a double-blind trial. This means that both you and your physician will not know which group you are in.

Why take part?

Patients often report fewer side-effects and better pain control with buprenorphine, but it is not yet known whether either medication is more effective. We cannot guarantee or promise that you will receive any benefits from this research. The outcome of your surgery will not be affected if you choose not to participate in the study.

Who can take part?

- Receiving pelvic exenteration surgery
- Age 18 years or more
- Your doctor thinks the trial is suitable for you

Are there costs to participate?

- There are no additional costs to you
- You will receive a gift card of $150 to reimburse you for your time

What should I expect?

Before surgery

- Phone call 15-30 minutes

During your hospital stay

- You will receive either oxycodone or buprenorphine for pain control after surgery
- These will be given to you only when you request it and used for up to 7 days
- Speak to a researcher for a few minutes at 1 week and 2 weeks after surgery about your pain experience

Day 90

- Phone call 30-60 minutes

> We will give you an information sheet that has more details about the **PROSPER** trial. Participation is voluntary.

PROSPER

Version number: 2. Date: 22-JAN-2026
