# Supplementary material for: Oral oxycodone versus sublingual buprenorphine for postoperative pain control after pelvic exenteration (PROSPER): a pilot, registry-embedded, multi-centre, double-blind, placebo-controlled, randomised controlled trial
Source: BMJ Open. 2026 Jun 22;16(6):e117594. doi: 10.1136/bmjopen-2026-117594 (PMC13289154; doi:10.1136/bmjopen-2026-117594)
Supplement: online supplemental file 2 [file bmjopen-16-6-s002.pdf]

# Participant Information Sheet/Consent Form

**Interventional Study** - *Adult providing own consent*

| | |
|---|---|
| **Title** | A **P**ilot, **R**egistry-embedded, multi-centre, double-blind, placebo controlled, randomised controlled trial of oral **O**xycodone versus **S**ublingual buprenorphine for postoperative pain control after **P**elvic **E**xente**R**ation (PROSPER) |
| **Short Title** | **O**xycodone versus **S**ublingual buprenorphine |
| **Protocol Number** | 2025/ETH01058 |
| **Sites** | Royal Prince Alfred Hospital<br>Peter MacCallum Cancer Centre<br>Royal Adelaide Hospital |
| **Project Sponsor** | University of Sydney |
| **Coordinating Principal Investigator** | Prof. Asad Patanwala |

## Part 1 What does my participation involve?

### 1 Introduction

You are invited to take part in this research project because you will be receiving pelvic exenteration surgery. The research project is testing two different options for pain control after surgery. The two treatment options are oxycodone and buprenorphine, which are opioid medications.

This Participant Information Sheet/Consent Form tells you about the research project. It explains the tests and treatments involved. Knowing what is involved will help you decide if you want to take part in the research.

Please read this information carefully. Ask questions about anything that you don’t understand or want to know more about. Before deciding whether or not to take part, you might want to talk about it with a relative, friend or your local doctor.

Participation in this research is voluntary. If you don’t wish to take part, you don’t have to. You will receive the best possible care whether or not you take part.

If you decide you want to take part in the research project, you will be asked to sign the consent section. By signing it you are telling us that you:

- Understand what you have read
- Consent to take part in the research project
- Consent to have the tests and treatments that are described
- Consent to the use of your personal and health information as described.

You will be given a copy of this Participant Information and Consent Form to keep.

The investigators have no conflicts of interest to declare.

## 2 What is the purpose of this research?

This is a feasibility study, which means the aim is to determine if a future large trial is possible in people who require pelvic exenteration surgery. Pelvic exenteration is a major surgery. It is expected that people will have pain after this surgery. To manage this postoperative pain, opioid medications are used during hospitalisation. Opioids such as oxycodone are typically used, but these have side-effects that can prolong recovery after surgery. For example, people may have constipation due to slow recovery of the intestines. People may also develop tolerance to oxycodone, which means the effectiveness of the drug decreases over time. A new opioid called buprenorphine has a different mechanism of action and may reduce some of these side-effects. It is possible that by giving people buprenorphine instead of oxycodone, they will have less side-effects and recover quicker. This has not yet been proven and there are no trials comparing oxycodone and buprenorphine in people who have had a pelvic exenteration. This feasibility trial involves a small number of people is the first step as it helps determine if a large comparative clinical trial is possible in the future.

Medications, drugs and devices have to be approved for use by the Australian Federal Government. Oxycodone and buprenorphine are approved in Australia to treat pain, which is the same as the indication for use in this trial.

This study will be conducted under the Therapeutic Goods Administration (TGA) Clinical Trials Notification (CTN) Scheme. This allows the investigators to use this product for medical research purposes once the research has been assessed and approved by an authorised Human Research Ethics Committee (HREC).

The results of this research will be used by the study doctor Charlotte Johnstone to obtain a Doctor of Philosophy degree. This research has been initiated by the study investigator, Professor Asad Patanwala. The research has been funded by the Medical Research Future Fund Clinical Trials Activity Grant from the Australian Government. This research is being sponsored by the University of Sydney.

## 3 What does participation in this research involve?

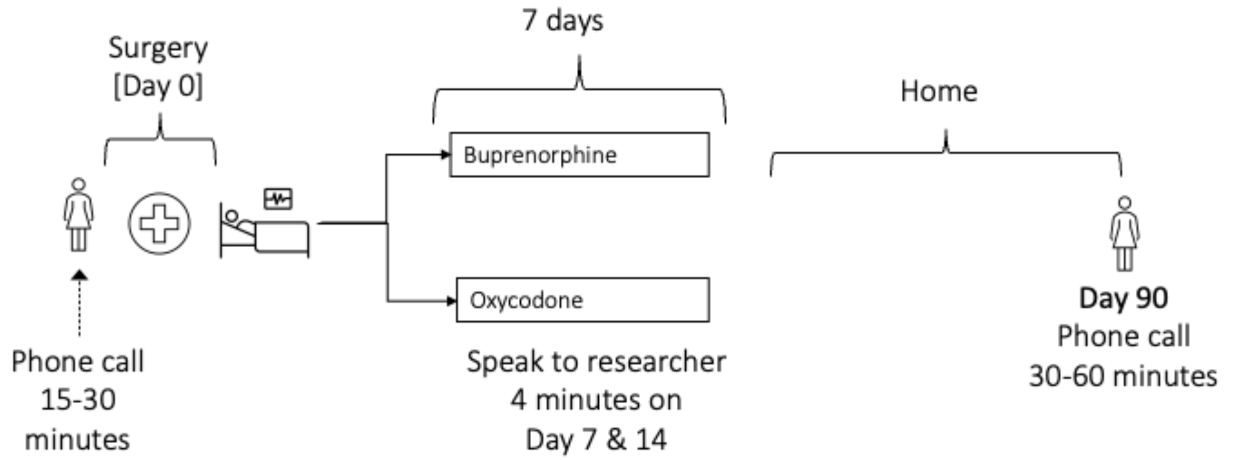

*Prior to surgery*
Your doctor has determined that you meet the criteria to participate in the study. Prior to your participation, you will need to sign a consent form. This must be done prior to your surgery to participate. After you have provided consent, we will obtain information about you related to your demographics (e.g., age, gender), medical history, pain medications, questionnaire regarding your pain (QoR-15), and quality of life (EQ-5D-5L). This can also be conducted during your regular clinic visit or over the phone. We anticipate that the discussion with the research staff to obtain this information from you will take 15 – 30 minutes.

*During hospitalisation*
You will be participating in a randomised controlled research project. Sometimes we do not know which treatment is best for treating a condition. To find out we need to compare different treatments. We put people into groups and give each group a different treatment. The results are compared to see if one is better. To try to make sure the groups are the same, each participant is put into a group by chance (random). You will be randomised to receive either oxycodone or buprenorphine. You have a one in two chance of receiving either of these options. The randomisation will take place after your surgery while you are in the hospital. You will be participating in a double-blind study, which means that you will not know which group you have been assigned.

Both oxycodone and buprenorphine are pain medications called opioids that are in tablet form. Oxycodone is a tablet that is swallowed, whereas buprenorphine is a tablet that dissolves under the tongue. These tablets are administered only if you need it depending on your pain level. Your doctor will decide after your surgery, how many tablets to give you each time you request medication for pain. You will get either 1 + 1 (1 tablet swallowed and 1 tablet under the tongue) or 2 + 2 (2 tablets swallowed and 2 tablets under the tongue). Whether you get 1+1 or 2+2 at each request will be determined by your doctor or nurse based on your pain. You will receive these tablets as often as every 3 hours when you need it for your pain. You will receive it only when you request you need it.

The first dose will be given to you after your doctor decides that you can take oral pain medicines after your surgery. If you do not have pain or have low amounts of pain, the medication is not used at that time until your pain increases again. These medications will be given to you up to 7 days starting after the first dose. After these 7 days, no more study medications will be given to you and your doctor will use any medications for your pain that they consider appropriate. If you do not participate in the trial, your doctor may still give you the same medications (oxycodone or buprenorphine) as part of your usual care.

During the first 14 days of your hospital stay, we will collect information from your medical records regarding your surgery, all pain medications you received, your pain level, recovery of your bowel, your mobility, and any adverse effects that you have had. We will directly obtain this from your medical records. There is nothing you need to do other than be aware that we are obtaining information from your medical records.

On day 7 and 14 after your surgery we will assess your recovery using the same questionnaire (QoR-15) we used before surgery. It takes approximately 4 minutes for the research staff to obtain this information from you.

*Day 90: After surgery*
On day 90 after your surgery, we will assess your recovery (QoR-15) and your quality of life (EQ-5D-5L) using the same questionnaires we used before your surgery. We will obtain your responses to the questionnaires over the phone. We will also obtain information from you about whether you are using pain medicines and if you have had any other medical visits or hospitalisations. We will also obtain information about your quality of life from a registry called PelvEx that will be routinely collecting data about your progress. Further information will be

collected from medical records regarding pain medicines used, doctor visits and hospitalisations.

During this call, we will interview you using a video call or over the phone. This call may be recorded. The interview is to ask you for your opinion about the trial. This includes if you found the study medications and the procedures of the trial to be acceptable and if you had any opinions or feedback to improve the study. The call on day 90 will take approximately 30 – 60 minutes.

## 4 Are these costs to me to participate?

There are no additional costs associated with participating in this research project. All medication, tests and medical care required as part of the research project will be provided to you free of charge. You will receive a gift card of $150 to reimburse you for the time taken to speak to the researchers. This will occur after your last follow-up phone call on day 90.

## 5 What do I have to do?

To participate in this study, you have to be willing to be interviewed by the researchers prior to surgery (15 – 30 minutes session), twice after surgery during your hospital stay (4 minutes each session), and once after hospital discharge on day 90 (30 – 60 minutes session). You also have to be willing to try the study drugs after your surgery as needed for your pain, which will be given to you for up to 7 days while you are in the hospital. It is only given to you when you need medication for pain. There are no other responsibilities or requirements.

## 6 Other relevant information about the research project

The study aims to recruit 30 participants overall across three hospitals. The study is a collaboration of researchers from the three hospital sites. This is a pilot feasibility study. This means that we will not be able to gauge from this study whether one drug is more effective or safer than the other. The aim is to determine if a larger study involving more participants is feasible.

## 7 Do I have to take part in this research project?

Participation in this research project is voluntary. If you do not wish to take part, you do not have to. If you decide to take part and later change your mind, you are free to withdraw from the project at any stage.

If you do decide to take part, you will be given this Participant Information and Consent Form to sign and you will be given a copy to keep.

Your decision whether to take part or not to take part, or to take part and then withdraw, will not affect your routine treatment, your relationship with those treating you or your relationship with the Royal Prince Alfred Hospital, Peter MacCallum Cancer Centre, or Royal Adelaide Hospital.

## 8 What are the alternatives to participation?

You do not have to take part in this research project to receive treatment at this hospital. If you decide not to participate, you will still receive pain management after your surgery. The pain management may include the same medications used in this study. This is because oxycodone or buprenorphine may or may not be used as part of usual treatment. In other words, if you choose not to participate, it is possible that the medications you receive may be the same as what you would receive during the study.

## 9 What are the possible benefits of taking part?

We cannot guarantee or promise that you will receive any benefits from this research; however, possible benefits may include improved recovery in participants who receive buprenorphine. This means that participants may feel better and have less side-effects. It is not proven at this time if buprenorphine is superior to oxycodone or not.

## 10 What are the possible risks and disadvantages of taking part?

Medical treatments often cause side effects. You may have none, some or all of the effects listed below, and they may be mild, moderate or severe. If you have any of these side effects, or are worried about them, talk with your study doctor. Your study doctor will also be looking out for side effects. We do not expect your pain to be greater on either drug.

There may be side effects that the researchers do not expect or do not know about and that may be serious. Tell your study doctor immediately about any new or unusual symptoms that you get.

Many side effects go away shortly after treatment ends. However, sometimes side effects can be serious, long lasting or permanent. If a severe side effect or reaction occurs, your study doctor may need to stop your treatment. Your study doctor will discuss the best way of managing any side effects with you.

*Side effects from oxycodone or buprenorphine*

| Common (>1%)<br>More than 1 in 100 | Infrequent (0.1-1%)<br>1 in 100-1000 | Rare (<0.1%)<br>Less than 1 in 1000 |
|---|---|---|
| Nausea and vomiting, upset stomach, drowsiness, dizziness, headache, low blood pressure, itch, dry mouth, reduce size of pupil of eyes, trouble emptying your bladder, constipation | Slow or stop breathing, difficulty breathing, confusion, hallucinations, confusion, agitation, mood changes, tremor, visual disturbances, skin rash, low body temperature, low or fast heart rate, high blood pressure, painful tightening of bile ducts, intestinal blockage, liver damage, stiff muscles, muscle jerks, flushing, low libido or energy. | Disorder of low salt levels in the blood, life-threatening allergic reaction, seizure |

*Side effect specific to buprenorphine*
Buprenorphine may interfere with the effect of other opioid medications. In rare circumstance (less than 1 in 1000), participants could experience withdrawal from opioids, which can result in increased pain.

*Duration of side effects*
The side effects listed generally go away when the medication is stopped.

*Effects on unborn child or newborn baby*
Pelvic exenteration surgery involves removal of the uterus in females. Therefore, there is no possibility for effects on unborn child or newborn baby. There is no effect of opioids on reproduction in males in terms of an unborn child. In addition, the duration of the opioids used in this trial is only during hospitalisation.

*Psychological distress*
If you become upset or distressed as a result of your participation in the research, the study doctor will be able to arrange for counselling or other appropriate support per usual care at the hospital or clinic. Any counselling or support will be provided by qualified staff who are not members of the research project team. This counselling will be provided free of charge.

*Burden of participation*
The burden of participation includes the time taken for interviews with the research staff to obtain information from you. It also includes the need to take tablets that are swallowed or placed under the tongue.

## 11 What if new information arises during this research project?

Sometimes during the course of a research project, new information becomes available about the treatment that is being studied. If this happens, your study doctor will tell you about it and discuss with you whether you want to continue in the research project. If you decide to withdraw, your study doctor will arrange for your regular health care to continue. If you decide to continue in the research project you will be asked to sign an updated consent form.

Also, on receiving new information, your study doctor might consider it to be in your best interests to withdraw you from the research project. If this happens, he/she will explain the reasons and arrange for your regular health care to continue.

## 12 Can I have other treatments during this research project?

Participation in this research project will not affect any medications or treatments you have been taking for your condition or for other reasons. It is important to tell your study doctor and the study staff about any treatments or medications you may be taking, including over-the-counter medications, vitamins or herbal remedies, acupuncture or other alternative treatments. This information will be obtained from you prior to surgery. While you are in the hospital after surgery and after hospital discharge, your doctor will decide what medications you should take. Participation in this study, will not affect other treatments. It may also be necessary for you to take medication during the research project to address side effects or symptoms that you may have. There will not be a charge to you for this and will be part of your usual care in the hospital.

## 13 What if I withdraw from this research project?

If you decide to withdraw from the project, please notify a member of the research team before you withdraw. This notice will allow that person or the research supervisor to discuss any health risks or special requirements linked to withdrawing.

If you do withdraw your consent during the research project, the study doctor and relevant study staff will not collect additional personal information from you, although personal information already collected will be retained to ensure that the results of the research project can be

measured properly and to comply with law. You should be aware that data collected by the sponsor up to the time you withdraw will form part of the research project results. If you do not want them to do this, you must tell them at the time of your withdrawal.

### 14 Could this research project be stopped unexpectedly?

This research project may be stopped unexpectedly for a variety of reasons. These may include reasons such as:

- Unacceptable side effects
- The drug being shown not to be effective
- The drug being shown to work and not need further testing
- Decisions made by local regulatory/health authorities

### 15 What happens when the research project ends?

After the project ends and when the data has been analysed, we will provide you with information regarding the success of the project. This information will be sent to you via email. You will be provided with a summary of the results when the project is completed. This is expected to occur as long as one year after your surgery.

## Part 2 How is the research project being conducted?

### 16 What will happen to information about me?

By signing the consent form you consent to the study doctor and relevant research staff collecting and using personal information about you for the research project. Any information obtained in connection with this research project that can identify you will remain confidential. All information from the project collected on paper will be stored in a locked cabinet in an office on the premises of *[Name of institution]*, that is only accessible to the site research staff.

The information will also be entered into a web-based secure electronic system called Research Electronic Data Capture (REDCap) maintained by the University of Sydney. Identifiable information about you will be kept in a master code sheet that will include a code that represents you. All other documents will not have your identifiers. Instead, it will just have your assigned code. The identifiable information (master code sheet) about you will be kept separately from the non-identifiable information. Your information will only be used for the purpose of this research project, and it will only be disclosed with your permission, except as required by law. The consent you provide is to use the data for this project only. It will not be used for future research. The information will be stored in REDCap for 15 years. After it is confirmed that all paper documents have been scanned and uploaded to REDCap, the paper documents will be destroyed by the site principal investigator.

Information about you may be obtained from your health records held at this and other health services for the purpose of this research. By signing the consent form you agree to the study team accessing health records if they are relevant to your participation in this research project.

Your health records and any information obtained during the research project are subject to inspection (for the purpose of verifying the procedures and the data) by the relevant authorities, the institution relevant to this Participant Information Sheet, *[Name of institution]*, or as required by law. By signing the Consent Form, you authorise release of, or access to, this confidential information to the relevant study personnel and regulatory authorities as noted above.

It is anticipated that the results of this research project will be published and/or presented in a variety of forums. In any publication and/or presentation, information will be provided in such a way that you cannot be identified. The information will only be presented in summary form to maintain confidentiality.

Information about your participation in this research project may be recorded in your health records.

In accordance with relevant Australian privacy and other relevant laws, you have the right to request access to your information collected and stored by the research team. You also have the right to request that any information with which you disagree be corrected. Please contact the study team member named at the end of this document if you would like to access your information.

Any information obtained for the purpose of this research project that can identify you will be treated as confidential and securely stored. It will be disclosed only with your permission, or as required by law.

## 17 Complaints and compensation

If you suffer any injuries or complications as a result of this research project, you should contact the study team as soon as possible and you will be assisted with arranging appropriate medical treatment. If you are eligible for Medicare, you can receive any medical treatment required to treat the injury or complication, free of charge, as a public patient in any Australian public hospital.

In addition, you may have a right to take legal action to obtain compensation for any injuries or complications resulting from the study. Compensation may be available if the injury or complication is sufficiently serious and is caused by unsafe drugs or equipment, or by the negligence of one of the parties involved in the study (for example, the researcher, the hospital, or the treating doctor). You do not give up any legal rights to compensation by participating in this study.

## 18 Who is organising and funding the research?

This research project is being conducted by Professor Asad Patanwala at the University of Sydney. No member of the research team will receive a personal financial benefit from your involvement in this research project (other than their ordinary wages).

## 19 Who has reviewed the research project?

All research in Australia involving humans is reviewed by an independent group of people called a Human Research Ethics Committee (HREC). The ethical aspects of this research project have been approved by the HREC of Sydney Local Health District (RPHA Zone).

This project will be carried out according to the *National Statement on Ethical Conduct in Human Research (2025)*. This statement has been developed to protect the interests of people who agree to participate in human research studies.

## 20 How is the study being monitored?

The research will be monitored by a study coordinator for quality assurance each month. This includes a double check to ensure that the data collected are accurate and the study is adhering to the protocol. Safety of the participants will be monitored by the principal investigator throughout the study, and selected serious adverse effects communicated to the investigators and Human Research Ethics committee.

## 21 Further information and who to contact

The person you may need to contact will depend on the nature of your query. If you want any further information concerning this project or if you have any medical problems which may be related to your involvement in the project (for example, any side effects), you can contact the principal study doctor *[Name of site PI]* on *[Phone of site PI]* or any of the following people:

**Clinical contact person**

| | |
|---|---|
| Name | *[Name of site PI]* |
| Position | *[Position of site PI]* |
| Telephone | *[Phone of site PI]* |
| Email | *[Email of site PI]* |

For matters relating to research at the site at which you are participating, the details of the local site complaints person are:

**Complaints contact person**

| | |
|---|---|
| Name | *[Name of site PI]* |
| Position | *[Position of site PI]* |
| Telephone | *[Phone of site PI]* |
| Email | *[Email of site PI]* |

**Coordinating investigator**

| | |
|---|---|
| Name | Prof. Asad Patanwala |
| Position | Chair of Clinical Pharmacy<br>Royal Prince Alfred Hospital \| University of Sydney |
| Telephone | 0437906443 |
| Email | Asad.patanwala@sydney.edu.au |

If you have any complaints about any aspect of the project, the way it is being conducted or any questions about being a research participant in general, then you may contact:

**Reviewing HREC approving this research and HREC Executive Officer details**

| | |
|---|---|
| Reviewing HREC name | Sydney Local Health District |
| HREC Executive Officer | Merela Ghazal |
| Telephone | 02 9515 6766 |
| Email | SLHD-RPAEthics@health.nsw.gov.au |
| Protocol Number | 2025/ETH01058 |

# **Consent Form -** *Adult providing own consent*

| | |
|---|---|
| **Title** | A **P**ilot, **R**egistry-embedded, multi-centre, double-blind, placebo controlled, randomised controlled trial of oral **O**xycodone versus **S**ublingual buprenorphine for postoperative pain control after **P**elvic **E**xente**R**ation (PROSPER) |
| **Short Title** | **O**xycodone versus **S**ublingual buprenorphine |
| **Protocol Number** | 2025/ETH01058 |
| **Project Sponsor** | University of Sydney |
| **Coordinating Principal Investigator** | Prof. Asad Patanwala |
| **Site Principal Investigator** | *[Enter site PI]* |
| **Location** | *[Enter hospital name]* |

## Declaration by Participant

I have read the Participant Information Sheet, or someone has read it to me in a language that I understand.

I understand the purposes, procedures and risks of the research described in the project.

I give permission for my doctors, other health professionals, hospitals or laboratories outside this hospital to release information to the University of Sydney concerning my disease and treatment for the purposes of this project. I understand that such information will remain confidential.

I have had an opportunity to ask questions, and I am satisfied with the answers I have received.

I freely agree to participate in this research project as described and understand that I am free to withdraw at any time during the study without affecting my future health care.

I understand that I will be given a signed copy of this document to keep.

I understand that, if I decide to discontinue the study treatment, a member of the research team may request my permission to obtain access to my medical records for collection of follow-up information for the purposes of research and analysis.

I understand that my participation in this study will allow the researchers and others, as described in the Information for Participants, to have access to my medical record, and I agree to this.

I understand that my de-identified data may be used for future research, and I agree to this.

I would like to receive a copy of the study results when they become available. My email address is: ________________

Note: All parties signing the consent section must date their own signature.

| | |
|---|---|
| Name of Participant (please print) _______________ | |
| Signature _______________ | Date _______________ |

| | |
|---|---|
| Name of Witness* to Participant's Signature (please print) _______________ | |
| Signature _______________ | Date _______________ |

* Needed if participant is unable to read per GCP/ICH E6(R3) 2.8.9. Witness is not to be the investigator, a member of the study team or their delegate. In the event that an interpreter is used, the interpreter may not act as a witness to the consent process. Witness must be 18 years or older.

**Declaration by Study Doctor/Senior Researcher†**

I have given a verbal explanation of the research project; its procedures and risks and I believe that the participant has understood that explanation.

| | |
|---|---|
| Name of Study Doctor/ Senior Researcher† (please print) _______________ | |
| Signature _______________ | Date _______________ |

† A senior member of the research team must provide the explanation of, and information concerning, the research project.
