# Supplementary material for: Oral oxycodone versus sublingual buprenorphine for postoperative pain control after pelvic exenteration (PROSPER): a pilot, registry-embedded, multi-centre, double-blind, placebo-controlled, randomised controlled trial
Source: BMJ Open. 2026 Jun 22;16(6):e117594. doi: 10.1136/bmjopen-2026-117594 (PMC13289154; doi:10.1136/bmjopen-2026-117594)
Supplement: online supplemental file 3 [file bmjopen-16-6-s003.docx]

**INTERVIEW GUIDE**

*The interview is part of the day 90 follow-up, which includes data acquisition for post-discharge outcomes, QoR-15, and EQ-5D-5L. The semi-structured interview is conducted after these data have been obtained.*

**Introduction**

- **Welcome**
  - "Thank you for agreeing to take part in this interview."
    - "Do you have any questions before we begin?"
    - "Are you happy for the interview to be recorded?"
- **Purpose and Framing**
  - "The purpose of today’s interview is to better understand your experience with pain and its management following surgery."
  - "There are no right or wrong answers. We’re interested in your honest thoughts and personal perspective."
- **Confidentiality and Process**
  - "Everything you share will be kept anonymous and will only be used for research purposes."
  - "This interview will take approximately 30 minutes. If you are becoming tired or would like to stop at any time, please let me know.”

**Section 1: Overall Experience**

- **Opening Question**
  - "To start, could you tell me about your overall experience with your recent surgery?"

*Prompt:* "Anything else you’d like to add about that experience?"

*Possible prompt:* what went well

*Possible prompt:* what didn’t go so well

- **Current State**
  - "Considering your surgery 3 months ago, how comfortable are you now?"
  - "If you're experiencing pain now, where is it?"

**Section 2: Preoperative Expectations**

- **Prior to Surgery**
  - "Before your surgery, what concerns—if any—did you have?"

*Prompts:*

- - Risk of complications?
  - Length of hospital stay?
  - Recovery period?
  - Managing physical changes?
  - Pain management?
- **Information Received**
  - "What information were you given about how your postoperative pain would be managed?"

*Prompts:*

- - Who provided it?
  - Did it meet your needs?
  - Did you have concerns about pain after surgery?

**Section 3: Postoperative Pain Experience**

- **Immediate Postoperative Period**
  - "Reflecting on the period right after your surgery, how well did the pain medication meet your expectations?"
- **Pain Management Experience**
  - "What treatments, including medications or therapies, were used to manage your pain?"
  - "What aspects of the pain management worked well?"
  - "What did not work well?"
- **Support and Barriers**
  - "What factors helped with your pain management? (e.g., family support, physiotherapy)"
  - "What barriers did you face in achieving better pain relief or comfort?"
- **Comparison Over Time**
  - "What was your pain like before surgery, immediately after surgery, and after you went home?"
  - "Overall, how would you compare your comfort now with how you felt before surgery?"

**Section 4: Clinical Trial Experience**

- **Trial Participation**
  - "Thinking about your involvement in the pain management trial:"
    - "How did you find the recruitment process?"
    - "Was obtaining analgesia through the trial straightforward?"
    - "Were your concerns addressed?"
    - "What aspects of the trial went well?"
    - "What could be improved?"

**Section 5: Broader Reflections**

- **Other Concerns and Suggestions**
  - "Considering the major surgery you’ve had, what are your main concerns now?
  - "Do you have any other thoughts or suggestions about how pain was managed after your surgery?"
  - "Is there anything else you'd like to share about your pain experience?"

**Conclusion**

- **Summary and Follow-up**
  - "Thank you for sharing your experience."
  - "If you have any further thoughts or questions, you’re welcome to contact us."
  - "Thanks again for your time and contribution to this research."
