# Supplementary material for: Oral oxycodone versus sublingual buprenorphine for postoperative pain control after pelvic exenteration (PROSPER): a pilot, registry-embedded, multi-centre, double-blind, placebo-controlled, randomised controlled trial
Source: BMJ Open. 2026 Jun 22;16(6):e117594. doi: 10.1136/bmjopen-2026-117594 (PMC13289154; doi:10.1136/bmjopen-2026-117594)
Supplement: Supplementary file 5 [file bmjopen-2026-117594.reviewer_comments.pdf]

PEER REVIEW HISTORY

BMJ Open publishes all reviews undertaken for accepted manuscripts. Reviewers are asked to complete a checklist review form and are provided with free text boxes to elaborate on their assessment. These free text comments are reproduced below.

ARTICLE DETAILS

Title (Provisional)

A pilot, registry-embedded, multi-centre, double-blind, placebo-controlled, randomised controlled trial of oral oxycodone versus sublingual buprenorphine for postoperative pain control after pelvic exenteration (PROSPER)

Authors

Johnstone, Charlotte; Koh, Cherry; Mathieson, Stephanie; Steffens, Daniel; White , Kate; Gray, Paul; Harris, Craig; Heriot, Alexander; Kramer , Kaitlin; Law, Chi Kin; Lin, Chung-Wei Christine; Liu, Xiaoqiu; Machado, Gustavo; McLachlan, Andrew; Penm, Jonathan; Riedel, Bernhard; Sammour, Tarik; Sanders, Robert; Solomon, Michael; Whitehead, Lilian; Winter, Des; Young, Jamie; Patanwala, Asad E

VERSION 1 - REVIEW

**Reviewer**	**1**
**Name**	**Komori, Koji**
**Affiliation**	**Aichi Cancer Center**
**Date**	**24-Feb-2026**
**COI**	**None**

Very good!

**Reviewer**	**2**
**Name**	**Zuo, Yunxia**
**Affiliation**	**Sichuan University, Anesthesiology**
**Date**	**28-Mar-2026**
**COI**	**None**

This is a protocol for a pilot study to investigate the feasibility of a full-scale randomised controlled trial to compare sublingual buprenorphine versus oral oxycodone for postoperative pain treatment following pelvic exenteration. The PICOS of this pilot study is well-described. The major concerns are:

1. The primary outcome of this pilot study is the rate of recruitment over a 6-month period. The authors didn't explain in detail why the rate of recruitment is important. The authors also didn't explain what's the following plan would be if the goal of recruitment was not reached. Instead, some descriptions in the Statistical Analysis Plan section appear more appropriate to mention in the introduction and discussion

2. This is a pilot study protocol for a full-scale trial. Some key issues related to the full-scale trial are not formally addressed. For example, what is the study design of trial? Is it a non-inferiority study or superiority study? What is the primary outcome of the full scale trial? The authors mentioned somewhere that QoR 15 would be the primary outcome. The rationale to use the rate of recruitment instead of QoR 15 as primary outcome for the pilot study needs to clarify.

3. In the introduction, the disadvantages of opioids use for postoperative pain management are emphasized. Does both oxycodone and buprenorphine belong to opioid category?

The minor concerns are:

1. The intervention requires greater detail and justification.

The trial compares: oral oxycodone 5–10 mg up to 3-hourly as required, versus sublingual buprenorphine 200–400 mcg 3-hourly as required. At present, the rationale for these dose ranges and the dosing equivalence between groups are insufficiently explained. More detail is needed regarding: the justification for the selected doses, whether these regimens are expected to be broadly equianalgesic, dose escalation rules, maximum daily doses, criteria for use of rescue medication, and the precise operational definition of "as required" in the postoperative setting. These details are especially important in a trial where analgesic exposure is central to feasibility and future trial planning.

2. Typographical Error (Line 197): Please remove the redundant parenthesis and period at the end of the sentence: "...as required (with oral placebo).)."

VERSION 1 - AUTHOR RESPONSE

Reviewer 2 comment 1
The primary outcome of this pilot study is the rate of recruitment over a 6-month period. The authors didn't explain in detail why the rate of recruitment is important.

<u>Response:</u> A description of why the rate of recruitment is important has been added to the text as suggested (Line 395-398): "The rate of recruitment is important for this trial as it would ensure that an adequate sample size can be achieved for a definitive trial based on the number of pelvic exenterations conducted across the included hospitals in Australia. This rationale is further detailed in the sample size section."

Reviewer 2 comment 2

The authors also didn't explain what the following plan would be if the goal of recruitment was not reached. Instead, some descriptions in the Statistical Analysis Plan section appear more appropriate to mention in the introduction and discussion

Response: If adequate recruitment does not occur than that provides us with information, we would need regarding the feasibility of a future definitive trial. This is the intent of our feasibility trial. The following text has been added to the discussion (Line 546-549): "Our primary outcome of recruitment rate is important to ensure the feasibility of a future definitive trial. If the recruitment is slower than anticipated, we would gain insight about this for future investigations in this population."

Reviewer 2 comment 3
This is a pilot study protocol for a full-scale trial. Some key issues related to the full-scale trial are not formally addressed. For example, what is the study design of the trial? Is it a non-inferiority study or a superiority study?

Response: The future full-scale trial will be a superiority study. This detail has been added to the sample size section. Text clarified (Line 479): "This will be a superiority study. Based on the minimal clinically important difference (MCID) on this instrument (MID = 8)[34], common standard deviation (SD = 18), we estimated that the full-scale trial would need 180 participants, based on a two-sided alpha of 0.05 and a power of 80%, considering 10% dropouts. "

Reviewer 2 comment 4
The authors mentioned that QoR 15 would be the primary outcome. The rationale for using the rate of recruitment instead of QoR 15 as the primary outcome for the pilot study needs to be clarified.

Response: The rate of recruitment was chosen as the primary outcome for this pilot trial because this aligns with the CONSORT guidelines for the outcomes used for pilot and feasibility trials (PMID: 27965879. Eldridge et al. CONSORT 2010 statement: extension to randomised pilot and feasibility trials). It is also the feasibility outcome that is the most important to determine progression to a future definitive trial. Pilot trials are not powered to evaluate differences in clinical outcomes that may be used in the future definitive trial. However, we have included the fidelity of data collection of our clinical outcomes such as QoR-15 as part of our secondary feasibility outcomes.

Reviewer 2 comment 5
In the introduction, the disadvantages of opioid use for postoperative pain management are emphasized. Does both oxycodone and buprenorphine belong to opioid category

Response: Yes, both oxycodone and buprenorphine are opioids. This information is in the introduction, where we have clarified some of the differences in mechanism between these opioids in the following paragraph (Line 264-277): "It is not clear which oral or sublingual opioid analgesic agent provides the best analgesia to adverse effect profile in the postoperative period. Oxycodone is a commonly prescribed opioid analgesic agent in

the postoperative period in Australia, accounting for more than 80% of opioids prescribed for some procedures[21]. One potential alternative strategy is to consider low-dose sublingual buprenorphine. In addition to being a opioid receptor full agonist, buprenorphine is a kappa receptor partial agonist[22]. In terms of analgesia, buprenorphine behaves as a full agonist and provides analgesia equivalent to other opioids such as morphine and fentanyl[23]. Buprenorphine may help reduce hyperalgesia because opioid-induced hyperalgesia is partly driven by dynorphins activating kappa opioid receptors, and buprenorphine's antagonism at these receptors can counteract this effect. This mechanism may also help reduce opioid tolerance, as shown in a pre-clinical model[24]. The partial agonism seen in vitro with buprenorphine may be beneficial for gastrointestinal activity. The sublingual presentation of buprenorphine has further for patients who are nil-by-mouth."

Reviewer 2 comment 6
The intervention requires greater detail and justification.
The trial compares oral oxycodone 5–10 mg up to 3-hourly as required, versus sublingual buprenorphine 200–400 mcg 3-hourly as required. At present, the rationale for these dose ranges and the dosing equivalence between groups is insufficiently explained. More detail is needed regarding: The justification for the selected doses

<u>Response</u>: We had added some text to justify this (Line 354-358): "These doses and time intervals are based on the investigators' clinical experience with this cohort of patients and what was being used across the hospitals in routine practice. The doses selected are also considered to be equianalgesic as defined by the Faculty of Pain Medicine, Australian and New Zealand College of Anaesthetists opioid conversion ratios[26]."

Reviewer 2 comment 6
More detail is needed regarding whether these regimens are expected to be broadly equianalgesic

<u>Response</u>: These are considered equianalgesic. The following text has been added (Line 356-358): "The doses selected are also considered to be equianalgesic as defined by the Faculty of Pain Medicine, Australian and New Zealand College of Anaesthetists opioid conversion ratios[26]."

Reviewer 2 comment 7
More detail is needed regarding dose escalation rules.

<u>Response</u>: Text has been added regarding escalation rules (Line 362-365): "Analgesia management may be escalated as per clinical need and usual practice. Pain is measured by the nursing staff through pain scores or functional activity. For example, as required parenteral analgesia such as subcutaneous morphine may be prescribed for severe pain that is not managed with the trial medication."

Reviewer 2 comment 8
More detail is needed regarding: Maximum daily doses

<u>Response</u>: Text has been added to clarify this (Line 348-349): "The amount of intervention medication that may be administered is 16 tablets per 24 hours."

Reviewer 2 comment 9
More detail is needed regarding the criteria for the use of rescue medication.

<u>Response</u>: We have added some text to clarify this (line 362-365): "Analgesia management may be escalated as per clinical need and usual practice. Pain is measured by the nursing staff through pain scores or functional activity. For example, as required parenteral analgesia such as subcutaneous morphine may be prescribed for severe pain that is not managed with the trial medication."

Reviewer 2 comment 10
More detail is needed regarding the precise operational definition of "as required" in the postoperative setting.

<u>Response</u>: We have added this operational definition as suggested (Line 352-343): "The term "as required" means that doses will be administered only when patients request it for pain control."

Reviewer 2 comment 11
Typographical Error (Line 197): Please remove the redundant parenthesis and period at the end of the sentence: "...as required (with oral placebo).)."

<u>Response</u>: The typographical error has been amended.
